# Changes in suicide in California from 2017 to 2021: a population-based study

**DOI:** 10.1186/s40621-023-00429-6

**Published:** 2023-03-27

**Authors:** Julia J. Lund, Elizabeth Tomsich, Julia P. Schleimer, Veronica A. Pear

**Affiliations:** Department of Emergency Medicine, University of California Davis School of Medicine, Sacramento, CA USA

**Keywords:** Self-harm, Suicide, Health disparities, COVID-19, Gun violence, Firearms

## Abstract

**Background:**

Suicide is a major public health problem with immediate and long-term effects on individuals, families, and communities. In 2020 and 2021, stressors wrought by the COVID-19 pandemic, stay-at-home mandates, economic turmoil, social unrest, and growing inequality likely modified risk for self-harm. The coinciding surge in firearm purchasing may have increased risk for firearm suicide. In this study, we examined changes in counts and rates of suicide in California across sociodemographic groups during the first two years of the COVID-19 pandemic relative to prior years.

**Methods:**

We used California-wide death data to summarize suicide and firearm suicide across race/ethnicity, age, education, gender, and urbanicity. We compared case counts and rates in 2020 and 2021 with 2017–2019 averages.

**Results:**

Suicide decreased overall in 2020 (4123 deaths; 10.5 per 100,000) and 2021 (4104; 10.4 per 100,000), compared to pre-pandemic (4484; 11.4 per 100,000). The decrease in counts was driven largely by males, white, and middle-aged Californians. Conversely, Black Californians and young people (age 10 to 19) experienced increased burden and rates of suicide. Firearm suicide also decreased following the onset of the pandemic, but relatively less than overall suicide; as a result, the proportion of suicides that involved a firearm increased (from 36.1% pre-pandemic to 37.6% in 2020 and 38.1% in 2021). Females, people aged 20 to 29, and Black Californians had the largest increase in the likelihood of using a firearm in suicide following the onset of the pandemic. The proportion of suicides that involved a firearm in 2020 and 2021 decreased in rural areas compared to prior years, while there were modest increases in urban areas.

**Conclusions:**

The COVID-19 pandemic and co-occurring stressors coincided with heterogeneous changes in risk of suicide across the California population. Marginalized racial groups and younger people experienced increased risk for suicide, particularly involving a firearm. Public health intervention and policy action are necessary to prevent fatal self-harm injuries and reduce related inequities.

**Supplementary Information:**

The online version contains supplementary material available at 10.1186/s40621-023-00429-6.

## Background

Suicide is significant public health problem and a leading cause of death in the United States (USA). From 2010 to 2020, more than 480,000 people nationally died by suicide, the majority by firearm suicide. Recent social, economic, and environmental stressors may have affected the burden of and disparities in suicide.

On March 19, 2020, California went under a stay-at-home order in response to the COVID-19 pandemic (Office of Governor Gavin Newsom 2020). Non-essential businesses, such as bars, fitness clubs, and some stores, were ordered to close, and residents were asked to “shelter-in-place” at home. Many people lost employment or had to leave jobs to caretake for children or family members. A high percentage of adults had trouble paying bills or rent due to the pandemic (Pew Research Center 2020), and most became more socially isolated (Abelson 2021). At the same time, hundreds of thousands experienced loss of a loved one to COVID-19 (Verdery et al. 2020).

Compounded with the pandemic and its repercussions were a variety of other co-occurring stressors in 2020 and 2021, including the widely publicized murder of George Floyd, subsequent protests and incidents of police brutality, political turmoil surrounding the 2020 presidential election, climate-change-fueled megafires, and collective grief and trauma resulting from these events (Silver et al. 2021; Bühler et al. 2022). Notably, not all groups were equally impacted. Marginalized communities and racialized groups bore the disproportionate burden of the social, health, and economic consequences of 2020 and 2021 (Wilson 2020) as a result of the ongoing legacy of structural racism in the USA, which has concentrated disadvantages (including poverty, under-funded schools, unemployment, over-policing and police violence, mass incarceration, and limited access to affordable healthcare, housing, and green spaces) among Black, Indigenous, and Hispanic communities (Bailey et al. 2017).

Financial difficulties, unemployment, social isolation, and trauma have all been linked to suicide and related risk factors (e.g., suicidality, depression) (Batty et al. 2018; Elbogen et al. 2020). One analysis found that in the years following the economic downturn from 2007 to 2009, an estimated 4750 more Americans died by suicide than projected (Reeves et al. 2012). A study of quarantined people during the severe acute respiratory syndrome (SARS) outbreak in 2003 found that approximately one-third of individuals experienced symptoms of depression and posttraumatic stress disorder (PTSD), with higher rates of PTSD symptoms associated with longer durations of quarantine (Hawryluck et al. 2004). Prior pandemics have also been linked to suicide: some research suggests that deaths by suicide increased overall during the 1918–1919 influenza pandemic in the USA (Wasserman 1992) and among older people aged 65 and above in Hong Kong during the SARS epidemic (Cheung et al. 2008).

Several studies have examined the link between the COVID-19 pandemic and suicide, but findings are mixed. One 2021 survey of adults in the USA found an association between COVID-19-related experiences (i.e., general distress, fear of infection, effects of social distancing policies) and increased suicidal ideation and nonfatal suicide attempts, with a substantial proportion of those reporting suicidal ideation explicitly attributing it to COVID-19 (Ammerman et al. 2021). Risk of self-harm has also been associated with high perceived stress due to COVID-19 (Caballero-Domínguez et al. 2022). However, a 2021 systematic review of time series analyses in Brazil, China, Ecuador, Mexico, Peru, Russian Federation, and Sri Lanka found no change in intentional self-harm during COVID-19 (Knipe et al. 2022). In the USA, two studies in California found no change in intentional drug-related overdoses (Kiang et al. 2022) or suicidal ingestions reported to the California Poison Control system (Ontiveros et al. 2021) following the pandemic. At the same time, there is evidence that—contrary to expectations—deaths by suicide decreased in Cook County, Illinois and in four Texas counties through July 31, 2020 (Pirkis et al. 2021). Differences between studies may stem in part from the populations under study. Importantly, analyses of aggregate trends may mask substantial heterogeneity in population subgroups, especially for subpopulations who comprise a minority of the overall population (Fox et al. 2022).

Beginning in 2020, firearm and ammunition purchasing in the USA far surpassed expected levels. Through July 2020, there were an estimated 4.3 million excess firearm purchases nationally (Schleimer et al. 2021) and, in California, approximately 110,000 people acquired a firearm and 390,000 purchased ammunition in response to the pandemic (Kravitz-Wirtz et al. 2021). Nationally, new firearm purchasers in 2020 and 2021 were more likely to be female, Black, or Hispanic (Miller et al. 2022). Handgun acquisition has been associated with large increases in firearm suicide risk, with a hazard ratio of nearly 8 among men and over 35 among women (Studdert et al. 2020). Despite this purchasing surge and the high lethality of firearm suicide attempts (Conner et al. 2019; Spitzer et al. 2020), few studies have examined pandemic-era trends in firearm suicide specifically, and none have looked at California, the most populous and diverse state in the USA.

The current study examines deaths by suicide and deaths by firearm suicide from 2017 through 2021 in California. Our aim is to assess changes in suicide during the first two years of the COVID-19 pandemic and determine whether changes varied by sociodemographic groups. We assess both counts and rates to evaluate different dimensions of the problem. Counts indicate population burden by identifying groups with the highest number of suicide deaths, while rates allow for between-group comparisons and reveal disparities in how different groups experience suicide.

## Methods

### Data

We used publicly available data on suicide from the California Department of Public Health – Vital Records Data (Cal-ViDa) query tool. The data contain statewide counts of deaths that occurred in California from January 1, 2017 to December 31, 2021, including information on decedents’ race and/or ethnicity (non-Hispanic white; non-Hispanic Black; non-Hispanic Asian; non-Hispanic Native American or Alaskan Native [AI/AN]; non-Hispanic Native Hawaiian or Pacific Islander [NH/PI]; Hispanic; or Other, which includes multi-race, other, and unknown), education level (Bachelor’s degree and higher or less than a Bachelor’s degree), gender identity (male or female), age (10–19, 20–29, 30–44, 45–64, 65+), county of residence (grouped by urbanicity according to the US Department of Agriculture’s Rural-Urban Continuum Codes [RUCCs], displayed in Additional file 1: Table S1), and cause of death, coded using the International Classification of Disease, 10th Revision (ICD-10) (State of California DoPH 2022). Deaths by suicide were defined using ICD-10 codes for intentional self-harm by discharge of firearms (X72-X74) and by other and unspecified means (*U03, X60-X71, X75-X84, Y87.0).

As in prior research, given the small number of cases coded as suicide among people under the age of 10 (Fatal Injury Reports, National, Regional, and States 2020), we restricted our sample to people aged 10 years and older. We used race and Hispanic ancestry/origin (race/ethnicity) as proxies for sociocultural differences which may modify risk for self-harm (Oquendo et al. 2005; Molock et al. 2006) and for the effects of interpersonal and structural racism, including historical redlining, residential and social segregation, punitive immigration policy, mass incarceration, and the concentration and transmission of intergenerational trauma (Bailey et al. 2017; Gee and Ford 2011; Forster et al. 2019; Morgan et al. 2022; Hawthorne et al. 2012; Owens et al. 2011; Primm et al. 2010), which are risk factors for self-harm.

### Analysis

We described rates and counts of fatal self-harm in California across our study period, comparing differences before and during the pandemic by method of suicide (firearm vs. any method) and across sociodemographic groups and geographic areas. To calculate suicide rates, we used estimates of the population aged 10 and older from the publicly available American Community Survey, 2017–2020 5-year estimates.

We compared monthly and annual counts and rates in 2020 and 2021 (which we refer to as “following the onset of the pandemic”) to the average of the 2017, 2018, and 2019 annual counts and rates (which we refer to as “pre-pandemic”). Only annual counts and rates were disaggregated by sociodemographic groups and geographic areas due to low monthly counts. To produce estimates for suppressed small numbers (Cal-ViDa data indicate “< 10” for all cell sizes 1–9), we used a single imputation technique developed for public health data which uses data in years prior to or after a missing cell to inform replacement, and mean imputation by year for the remaining missing values (Erdman et al. 2021). Up to 35% of Cal-ViDa observations had missing data (primarily due to suppressed county-level suicide counts in small counties). All statistical analyses were done using R version 4.1.2 (R Core Team 2020).

## Results

**Fig. 1:**
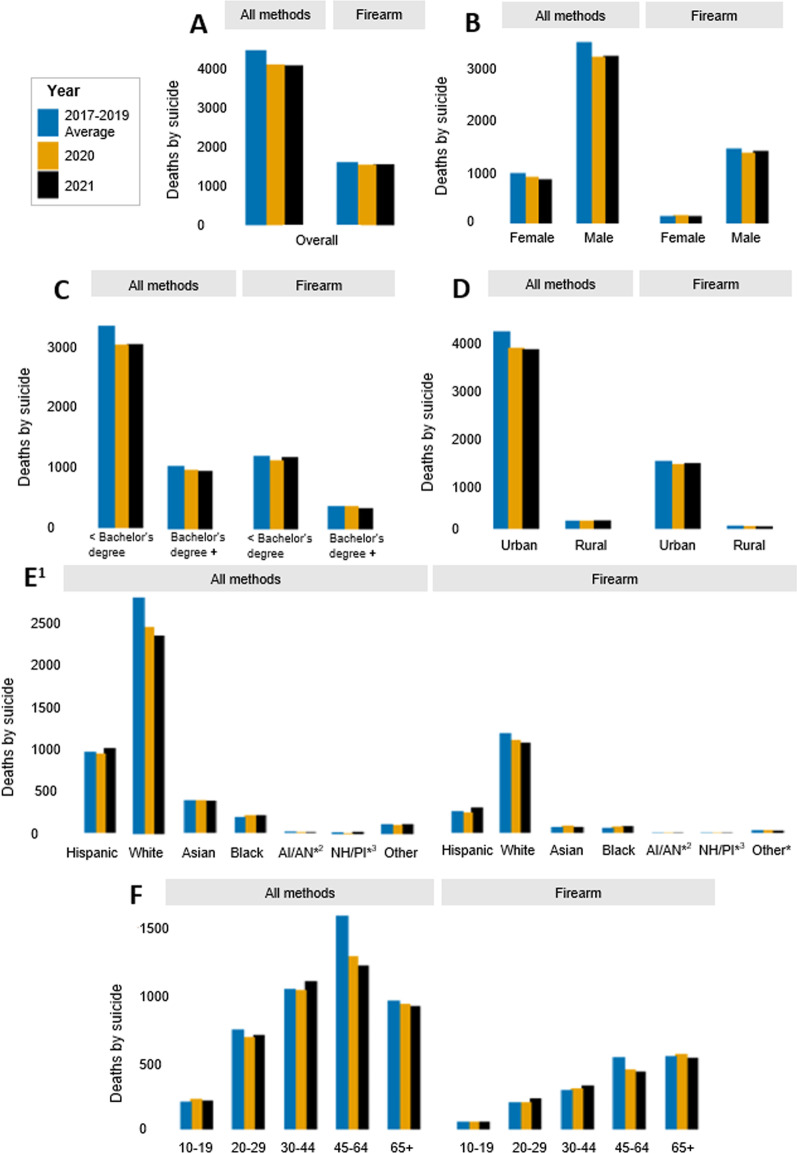
Counts of suicide in California from 2017 to 2021, by method of harm (**A**) in total population, and stratified by (**B**) sex (**C**) highest level of education (**D**) urbanicity (**E**) race/ethnicity and (**F**) age group. ^1^All race/ethnicity categories besides Hispanic and Other are non-Hispanic. ^2^AI/AN = American Indian (Native American)/Alaskan Native. ^3^NH/PI = Native Hawaiian/Pacific Islander. *Counts of 30 or less. **Note: Y-axis scales differ across panels

**Fig. 2:**
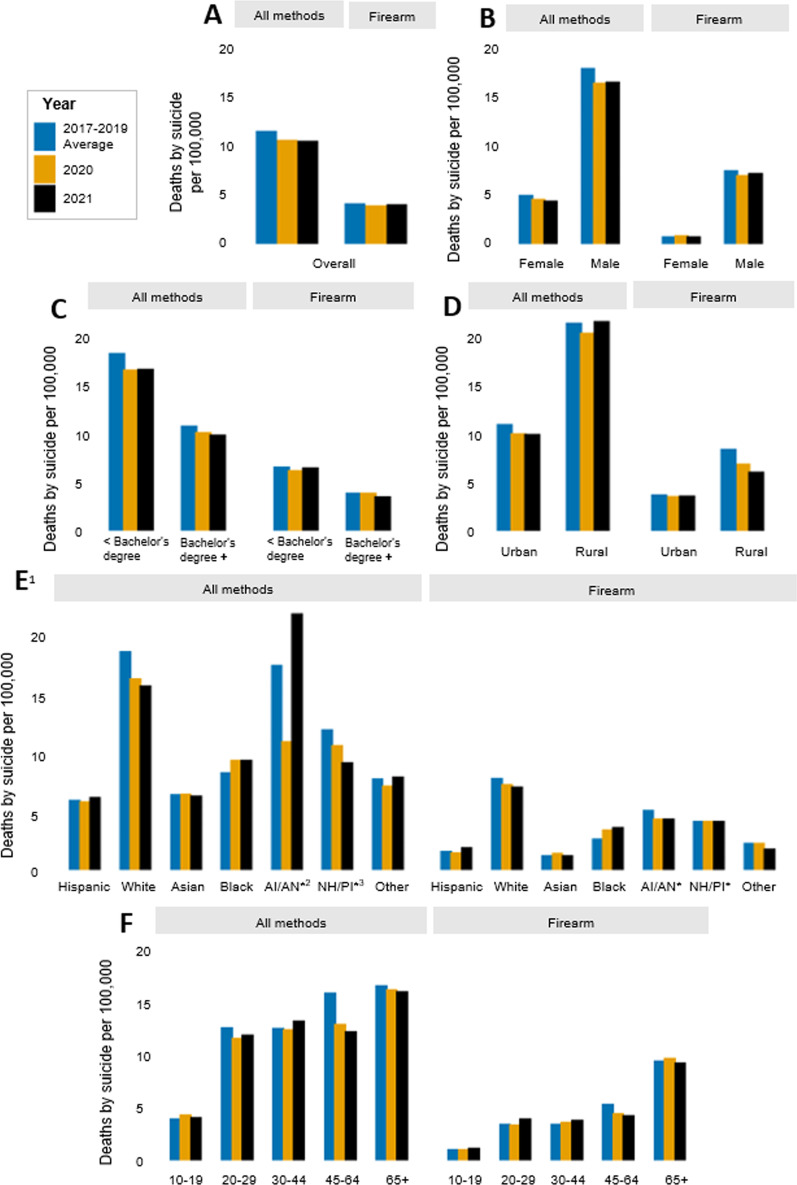
Rates of suicide in California from 2017 to 2021, by method of harm (**A**) in total population, and stratified by (**B**) sex (**C**) highest level of education (**D**) urbanicity (**E**) race/ethnicity and (**F**) age group. ^1^All race/ethnicity categories besides Hispanic and Other are non-Hispanic. ^2^AI/AN = American Indian (Native American)/Alaskan Native. ^3^NH/PI = Native Hawaiian/Pacific Islander. *Rate is based on counts of 30 or less

There was a total of 8227 suicides in California in the two years following the onset of the COVID-19 pandemic: 4123 (10.5 per 100,000) in 2020 and 4104 (10.4 per 100,000) in 2021 (Figs. 1A and 2A; Additional file 1: Tables S2 and S3). Pre-pandemic, the state-wide suicide burden was higher, with an average of 4484 deaths per year (11.4 per 100,000) in 2017–2019. Following the onset of the pandemic, there was also a slight decline in firearm suicides, with 1618 deaths (4.1 per 100,000) pre-pandemic, 1550 deaths (3.9 per 100,000) in 2020, and 1564 deaths (4.0 per 100,000) in 2021. Monthly trends in suicide and firearm suicide across the study period are given in Additional file 1: Fig. S1 and Additional file 1: Table S5. Because the decline in overall suicide was greater than the decline in firearm suicides, the proportion of suicides involving a firearm (36.1% pre-pandemic) increased slightly by 1.5% and 2.0%, in 2020 and 2021, respectively (Additional file 1: Table S4).

Across the study period, suicide and firearm suicide counts and rates were higher among males compared to females (Figs. 1B and 2B; Additional file 1: Tables S2 and S3). Males consistently represented about 78% of all suicides and about 90% of firearm suicides in California. In the total population, counts and rates of suicide decreased by approximately 8% in 2020 compared to pre-pandemic; however, in 2021, females experienced a greater decline (about 12% reduction) compared to pre-pandemic than did males (about 8% reduction). The burden of firearm suicide increased slightly among females in 2020 compared to pre-pandemic, with 14 more deaths from firearm suicide among females (an increase from 0.8 to 0.9 per 100,000), while the burden decreased among males that year, with 82 fewer firearm suicides (a reduction from 7.5 to 7.0 per 100,000). Consistently across the study period, firearm use in suicide was more common among males, with firearm suicides accounting for about 42–44% of all suicides among males and 16–19% of all suicides among females (Additional file 1: Table S4).

Rates and counts of suicide and firearm suicide varied by county urbanicity throughout the study period (Figs. 1D and 2D; Additional file 1: Tables S2 and S3). The highest number of suicides occurred in urban counties (metropolitan areas), but rural counties (nonmetropolitan areas) consistently had higher rates of suicide and firearm suicide. Compared to urban areas, rural areas saw a larger relative decrease in the proportion of suicides that involved a firearm in 2020 and 2021 compared to years prior (Additional file 1: Table S4).

The burden of and trends in suicide also differed across racial and ethnic groups (Figs. 1E and 2E; Additional file 1: Table S2 and S3). Non-Hispanic white Californians, who comprise 37% of the total population, had the highest number of suicides, accounting for over half of all suicides and firearm suicides in the state in all years of the study period. Hispanic Californians consistently accounted for the next largest number of suicides and firearm suicides, followed by Asian, Black, Other, AI/AN, and NH/PI Californians. Rates of suicide and firearm suicide were generally highest among white Californians, with AI/AN, NH/PI, and Black Californians experiencing the next highest rates. Hispanic and Asian Californians had the lowest rates of suicide and firearm suicide across the study period.

The state-wide decrease in suicide following the onset of the pandemic was not felt or distributed evenly across racial and ethnic groups. For instance, compared to pre-pandemic, non-Hispanic white Californians experienced 348 fewer suicide deaths (a reduction from 19.4 to 17.0 per 100,000) in 2020 and 445 fewer (a reduction from 19.4 to 16.4 per 100,000) in 2021. In contrast, Black Californians experienced 23 more deaths (an increase from 8.7 to 9.8 per 100,000) both in 2020 and 2021, compared to years prior. Though modest, Black Californians also had the highest and most stable increase in firearm suicides following the onset of the pandemic, with 17 more deaths (an increase from 2.8 to 3.6 per 100,000) in 2020 and 21 more deaths (an increase from 2.8 to 3.8 per 100,000) in 2021, compared to other racial/ethnic groups, all of whom experienced a decrease or no change in at least one of the years.

The burden of and changes in suicide and firearm suicide also varied by age group (Figs. 1F and 2F; Additional file 1: Table S2 and S3). In all years of the study period, Californians aged 30 to 64 accounted for the most suicide deaths (56–58%), while Californians aged 45 to 65+ accounted for the most firearm suicide deaths (61–66%). Rates were higher with increasing age, with people aged 10 to 19 having the lowest rates of suicide (4.0–4.4 per 100,000) and firearm suicide (1.1–1.2 per 100,000), and people aged 65 + having the highest rates of suicide (16.1–16.7 per 100,000) and firearm suicide (9.3–9.8 per 100,000). In 2020, however, young people aged 10 to 19 experienced 21 more suicides (an increase from 4.0 to 4.4 per 100,000) compared to pre-pandemic, while all other age groups saw a decline. The largest decline was among people aged 45–64, who had nearly 300 fewer suicides (a reduction from 16.0 to 13.0 per 100,000) in 2020 compared to pre-pandemic. People aged 65+ who died by suicide were consistently the most likely to use a firearm (Additional file 1: Table S4). Compared to pre-pandemic, the largest increase in the proportion of suicides that involved a firearm was among people aged 65+ in 2020 (from 57.0 to 60.2%) and among people aged 20 to 29 in 2021 (from 27.4 to 33.1%).

## Discussion

During the first two years following the onset of the COVID-19 pandemic, the incidence of suicide declined state-wide in California, while firearm suicide rates declined much more modestly. Differential variation by sociodemographic groups and geographic areas underlay these trends, suggesting differential exposure to or impact of pandemic-era risk and protective factors, and a need for tailored allocation of state resources and prevention efforts.

The overall decline in state-wide suicide rates during and following the onset of the pandemic parallels similar findings from other states (Faust et al. 2021; Mitchell and Li 2021; Bray et al. 2021), and the slight decline in firearm suicide rates aligns with recent CDC data released indicating firearm suicide rates remained level between 2019 and 2020 (Kegler et al. 2022). In California, the overall decline was driven by meaningful reductions in suicide among the groups most burdened by suicide: male, middle-aged, and white Californians. In contrast, groups typically at lower risk—female, young, Black, and Hispanic Californians—experienced increases or relatively smaller decreases in suicide or firearm suicide.

As in prior research, we found that males in California consistently had a higher risk of suicide (Callanan and Davis 2012). However, females experienced a slight increase in firearm suicide in 2020 compared to pre-pandemic, while males experienced a decrease. These results may reflect the fact that the recent firearm purchasing surge led to uniquely higher firearm ownership among groups historically less likely to own firearms (e.g., women) and may indicate a potential shift toward more lethal methods among this group (Miller et al. 2022).

Young people (ages 10 to 19) in California experienced an increase in suicide in 2020 and 2021 compared to years prior, a trend mirroring national findings (Kegler et al. 2022). This is especially concerning given that suicide is the second leading cause of death for young people in California and nationally (California Department Public Health 2018; CDC. CDC WONDER 2020). The magnitude of the increase among young people was not shared by any other age groups, most of whom experienced a decrease in suicide. Preventative efforts, including lethal means safety and mental health supports, should be prioritized for adolescents and young adults—who were uniquely impacted by recent social isolation, uncertainty, stress, and fear—given their stage of life and the importance of socialization for healthy development (Viner et al. 2022; United Nations 2020).

White Californians experienced substantial declines in suicide and firearm suicide during the pandemic, a trend aligned with national findings (Stone et al. 2023). Given the size of the white population and the magnitude of suicide burden among this group, this decrease drove the overall decline observed in the aggregated data. By disaggregating the data, we discovered unique trends across distinct communities. For instance, Black, Hispanic, Asian, and AI/AN Californians all experienced an increase in suicide and/or firearm suicide in at least one of the years following the onset of the pandemic. Compared to all other racial/ethnic groups, Black Californians experienced the largest relative increase in suicide and firearm suicide following the onset of the pandemic. These findings are consistent with studies in Maryland and Connecticut documenting an increase in suicide mortality among Black residents and a decrease among white residents in the months following the onset of the pandemic compared to earlier time periods (Mitchell and Li 2021; Bray et al. 2021), and with national, pre-pandemic trends showing a greater increase in suicidal behavior among Black Americans, particularly youth, compared to white Americans, from 1991 to 2019 (Xiao et al. 2021).

It is likely the racial/ethnic disparities we identified are related, in part, to the pandemic-driven amplification of the structural inequities that shape population health in the USA (Gravlee 2020) and diminshment of culturally specific factors protective of suicide. The communities most burdened by the health, economic, and social crises of 2020 and 2021 already faced disproportionate threats to their health as a result of systemic racism (Bailey et al. 2017) and other systems of marginalization that concentrate greater risk factors associated with suicide (e.g., poverty, unemployment, and mass incarceration (Gee and Ford 2011; Morgan et al. 2022)) and fewer protective factors (e.g., quality education, economic development, and culturally competent mental healthcare (Hawthorne et al. 2012; Owens et al. 2011; Primm et al. 2010)). Further, Black and Latino Americans, who attend church at higher rates than white Americans (Pew Research Center 2014), may have been disproportionately impacted by the restricted ability to gather for religious worship; and religiosity has been linked to reduction in suicide risk (Molock et al. 2006; Lawrence et al. 2016). In addition, COVID-19 increased economic and labor market disparities along racial lines (Wilson 2020), which have been connected to increased risk of suicide (Wadsworth and Kubrin 2007). Finally, perceived racial discrimination, which increased during the pandemic (Martínez-Alés et al. 2022; Wang et al. 2021), along with disparities in death from COVID-19 and police killings (Wilson 2020; Martínez-Alés et al. 2022), has also been connected to suicide risk among racially/ethnically minoritized groups (Wang et al. 2021; Mpofu et al. 2022).

Another factor potentially contributing to the increase in suicide, particularly firearm suicide, among some groups may be the firearm purchasing surge of 2020 and 2021. There is an established connection between firearm access and risk of firearm suicide (Studdert et al. 2020; Kellermann et al. 1992; Miller et al. 2002; Wintemute et al. 1999), and surges in firearm purchasing, which California experienced at the onset of the pandemic, are associated with increases in firearm violence (Levine and McKnight 2017; Laqueur et al. 2019). Further, a national study found that pandemic-era firearm purchasers were more likely to experience suicidality than non-owners and pre-pandemic purchasers (Anestis et al. 2021). While we did not observe an increase in number of firearm suicides following the onset of the pandemic, the increase in proportion of suicides that involve a firearm could indicate a trend toward an increasing use of firearms for self-harm, even amid an overall decreasing trend in death by suicide. Further, while nonurban areas saw a decrease in the proportion of suicides that involve a firearm, urban areas saw an increase. This may reflect the fact that the purchasing surge of 2020 and 2021 was especially pronounced in urban areas (Miller et al. 2022). Additionally, suicide modestly increased in rural areas in 2021, compared to pre-pandemic, while firearm suicide decreased, which may point to greater use of non-firearm means of suicide in California’s rural population—a finding which does not align with historical trends or findings from other contexts (Nestadt et al. 2017; Branas et al. 2004). Future studies with more granular data on method of suicide should explore these questions. Either way, investment in firearm violence prevention strategies—including education on safe storage practices and promotion of extreme risk protection orders (Pear et al. 2022)—may help reduce risk for firearm suicide.

For white populations and others who experienced a decline in suicide during the pandemic, a few potentially protective factors introduced during this period may have buffered or modified the expected association between the stressors of 2020 and 2021 and risk for suicide. For instance, a sense of shared experience may have offset the lack of social interaction by creating a feeling of collective purpose (Schaefer et al. 2013). In addition, people who lived with others during the stay-at-home order may have been alone less often or under higher levels of supervision or scrutiny within their home, which may have reduced self-harm. Reductions in in-person healthcare appointments at the start of the pandemic led to the widespread adoption of telehealth, which may have increased some individuals’ access to mental healthcare (Myers 2019). Finally, COVID-19 relief payments may have offset financial strain for some (Kim 2021). Each of these potentially protective factors is likely differential based on one’s access to remote work and other economic protections; non-Hispanic white and male Californians are relatively advantaged in both regards (Asfaw 2022), which could help explain the decline in suicide those groups experienced.

To our knowledge, this is the first study to assess incidence of firearm suicide across sociodemographic groups in California following the onset of the pandemic. There are, however, several limitations. First, due to data availability, we are only able to stratify deaths by suicide across one method (i.e., firearm). As such, we cannot pinpoint the method of suicide driving observed changes. Further, our inability to stratify death data across more than one domain restricts the nuance of our analyses. Future research should characterize risk across the intersection of multiple groups and compare changes in other methods of suicide.

## Conclusions

In California, the groups most burdened by suicide—males, middle-aged, and white Californians—experienced meaningful decreases in suicide following the onset of the pandemic, driving decreases in overall population rates, while females, young, Black, and Hispanic Californians experienced increases or small decreases, worsening existing health inequities. Identifying factors underlying these trends may inform our understanding of the epidemiology of suicide in various communities. Our findings highlight the need for targeted interventions addressing structural inequities, such as implementation of more social supports and provision of basic needs; suicide prevention interventions, such as improved access to quality mental healthcare, expansion of bereavement counseling, and better suicide risk assessments; and firearm safety efforts, such as trainings on safe storage practices and education about extreme risk protection orders, all of which could reduce suicide and promote equitable opportunity for health and wellness across the state.

## Supplementary Information

**Additional file 1**. **Figure S1**: Rates of suicide and firearm suicide in California from 2017–2021, by month. **Table S1**: California counties categorized as rural/urban according to the US Department of Agriculture’s Rural-Urban Continuum Codes (RUCCs). **Table S2**: Counts of suicide and firearm suicide in California from 2017–2021, by sociodemographic characteristics. **Table S3**: Rates of suicide and firearm suicide in California from 2017–2021, by sociodemographic characteristics. **Table S4**: Proportion of suicides that involved a firearm in California from 2017–2021, by sociodemographic characteristics. **Table S5**: Counts and rates of suicide and firearm suicide in California from 2017–2021, by month.

## Data Availability

The dataset of deaths in California generated and analyzed during the current study are publicly available in the California Department of Public Health – Vital Records Data (Cal-ViDa) query tool repository, https://cal-vida.cdph.ca.gov/ (State of California DoPH. California Vital Data (Cal-ViDa). 2022).

## Abbreviations

AI/AN: Native American or Alaskan Native
Cal-ViDA: California Department of Public Health – Vital Records Data
ICD-10: International Classification of Disease, 10th Revision
NH: Non-Hispanic
NH/PI: Native Hawaiian or Pacific Islander
PTSD: Posttraumatic stress disorder
SARS: Severe acute respiratory syndrome
UC: University of California
USA: United States of America
