# Supplementary material for: Changes in suicide in California from 2017 to 2021: a population-based study
Source: Inj Epidemiol. 2023 Mar 27;10:19. doi: 10.1186/s40621-023-00429-6 (PMC10041498; doi:10.1186/s40621-023-00429-6)
Supplement: Supplementary file 1 — Additional file 1. Figure S1: Rates of suicide and firearm suicide in California from 2017–2021, by month. Table S1: California counties categorized as rural/urban according to the US Department of Agriculture’s Rural-Urban Continuum Codes (RUCCs). Table S2: Counts of suicide and firearm suicide in California from 2017–2021, by sociodemographic characteristics. Table S3: Rates of suicide and firearm suicide in California from 2017–2021, by sociodemographic characteristics. Table S4: Proportion of suicides that involved a firearm in California from 2017–2021, by sociodemographic characteristics. Table S5: Counts and rates of suicide and firearm suicide in California from 2017–2021, by month. [file 40621_2023_429_MOESM1_ESM.docx]

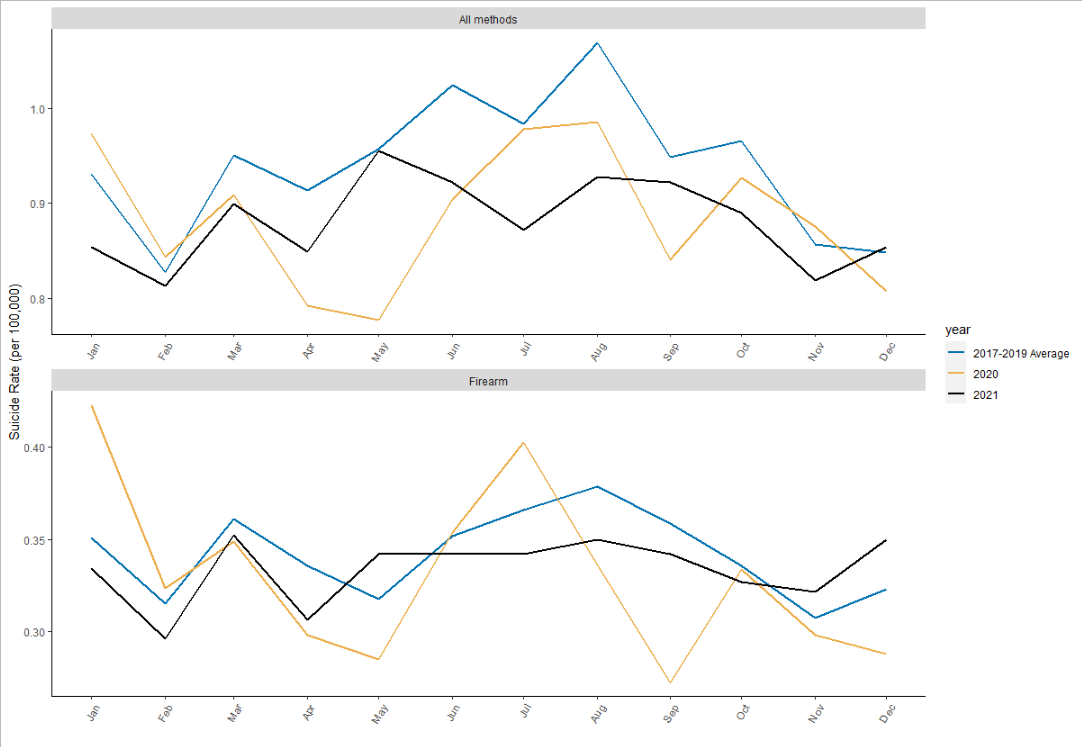


*Supplemental Figure 1. Rates of suicide and firearm suicide in California from 2017-2021, by month*

Supplemental Table 1. California counties categorized as rural/urban according to the US Department of Agriculture’s Rural-Urban Continuum Codes (RUCCs)

| RUCC Code | Description | names of Counties | Urban/Rural |
| --- | --- | --- | --- |
| 1 | Counties in metro areas of 1 million population or more | Alameda, Contra Costa, El Dorado, Los Angeles, Marin, Orange, Placer, Riverside, Sacramento, San Benito, San Bernardino, San Diego, San Francisco, San Mateo, Santa Clara, Yolo | \| Urban (metropolitan area)   37 counties \| \| --- \| \|  \| \|  \| |
| 2 | Counties in metro areas of 250,000 to 1 million population | Fresno, Kern, Merced, Monterey, San Joaquin, San Luis Obispo, Santa Barbara, Santa Cruz, Solano, Sonoma, Stanislaus, Tulare, Ventura |  |
| 3 | Counties in metro areas of fewer than 250,000 population | Butte, Imperial, Kings, Madera, Napa, Shasta, Sutter, Yuba |  |
| 4 | Urban population of 20,000 or more, adjacent to a metro area | Lake, Mendocino, Nevada, Tehama, Tuolumne | \| Rural (nonmetropolitan area)   21 counties \| \| --- \| \|  \| \|  \| \|  \| \|  \| |
| 5 | Urban population of 20,000 or more, not adjacent to a metro area | Humboldt |  |
| 6 | Urban population of 2,500 to 19,999, adjacent to a metro area | Amador, Calaveras, Colusa, Glenn, Modoc, Siskiyou |  |
| 7 | Urban population of 2,500 to 19,999, not adjacent to a metro area | Del Norte, Inyo, Lassen, Mono, Plumas |  |
| 8 | Completely rural or less than 2,500 urban population, adjacent to a metro area | Alpine, Mariposa, Sierra, Trinity |  |
| 9 | Completely rural or less than 2,500 urban population, not adjacent to a metro area | - |  |

Supplemental Table 2. Counts of suicide and firearm suicide in California from 2017-2021, by sociodemographic characteristics

|  |  | **All methods** | | | | |  | |  | **Firearm** | | | | |  | | |
| --- | --- | --- | --- | --- | --- | --- | --- | --- | --- | --- | --- | --- | --- | --- | --- | --- | --- |
|  | **Number of suicides** | | | | **Count change (% Change****^1^)** | | | **Number of suicides** | | | | | **Count change  (% Change^1^)** | | | |  |
|  | **2017-19 Average** | | **2020** | **2021** | **2017-19 average  to 2020** | **2017-19 average  to 2021** | | **2017-19 Average** | | | **2020** | **2021** | **2017-19 average  to 2020** | **2017-19 average  to 2021** | | |  |
| **STATE-WIDE** | 4484 | | 4123 | 4104 | -361  (-8.1%) | -380  (-8.5%) | | 1618 | | | 1550 | 1564 | -68  (-4.2%) | -54  (-3.3%) | | |  |
| **SEX** | | | | | | | | | | | | | | | |  | |
| Female | 983 | | 908 | 866 | -75  (-7.6%) | -117  (-11.9%) | | 160 | | | 174 | 155 | +14  (8.8%) | -5  (-3.1%) | | |  |
| Male | 3500 | | 3215 | 3237 | -285  (-8.1%) | -263  (-7.5%) | | 1458 | | | 1376 | 1409 | -82  (-5.6%) | -49  (-3.4%) | | |  |
| **AGE GROUP** | | | | | | | | | | | | | | | |  | |
| 10 to 19 | 204 | | 225 | 212 | +21  (10.3%) | +8  (3.9%) | | 59 | | | 58 | 60 | -1  (-1.7%) | +1  (1.7%) | | |  |
| 20 to 29 | 734 | | 674 | 691 | -60  (-8.2%) | -43  (-5.9%) | | 201 | | | 198 | 229 | -3  (-1.5%) | +28  (13.9%) | | |  |
| 30 to 44 | 1028 | | 1023 | 1085 | -5  (-0.5%) | +57  (5.5%) | | 289 | | | 302 | 321 | +13  (4.5%) | +32  (11.1%) | | |  |
| 45 to 64 | 1568 | | 1270 | 1202 | -298  (-19.0%) | -366  (-23.3%) | | 531 | | | 441 | 423 | -90  (-17.0%) | -108  (-20.3%) | | |  |
| 65+ | 945 | | 921 | 906 | -24  (-2.5%) | -39  (-4.1%) | | 539 | | | 554 | 527 | +15  (2.8%) | -12  (-2.2%) | | |  |
| **RACE/ETHNICITY** | | | | | | | | | | | | | | | |  | |
| Hispanic | 957 | | 940 | 1002 | -17  (-1.8%) | +45  (4.7%) | | 257 | | | 242 | 301 | -15  (-5.8%) | +44  (17.1%) | | |  |
| NH^2^ White | 2794 | | 2446 | 2349 | -348  (-12.5%) | -445  (-15.9%) | | 1180 | | | 1098 | 1066 | -82  (-7.0%) | -114  (-9.7%) | | |  |
| NH Asian | 387 | | 388 | 380 | +1  (0.3%) | -7  (-1.8%) | | 74 | | | 87 | 74 | +13  (17.6%) | 0  (0.0%) | | |  |
| NH Black | 187 | | 210 | 210 | +23  (12.3%) | +23  (12.3%) | | 61 | | | 78 | 82 | +17  (27.9%) | +21  (34.4%) | | |  |
| NH AI/AN^3^ | 24 | | 15 | 30 | -9  (-37.5%) | +6  (25.0%) | | 7 | | | 6 | 6 | -1  (-14.3%) | -1  (-14.3%) | | |  |
| NH NH/PI^4^ | 17 | | 15 | 13 | -2  (-11.8%) | -4  (-23.5%) | | 6 | | | 6 | 6 | 0  (0.0%) | 0  (0.0%) | | |  |
| Other | 117 | | 109 | 120 | -8  (-6.8%) | +3  (2.6%) | | 35 | | | 34 | 28 | -1  (-2.9%) | -7  (-20.0%) | | |  |
| **HIGHEST LEVEL OF EDUCATION** | | | | | | | | | | | | | | | |  | |
| Less than a Bachelor’s degree | 3322 | | 3018 | 3025 | -304  (-9.2%) | -297  (-8.9%) | | 1207 | | | 1134 | 1183 | -73  (-6.1%) | -24  (-2.0%) | | |  |
| Bachelor's Degree or higher | 1044 | | 979 | 954 | -65  (-6.2%) | -90  (-8.6%) | | 378 | | | 378 | 343 | 0  (0.0%) | -35  (-9.3%) | | |  |
| **URBANICITY** | | | | | | | | | | | | | | | |  | |
| Urban (metro) | 4275 | | 3921 | 3891 | -354  (-8.3%) | -384  (-9.0%) | | 1469 | | | 1406 | 1422 | -63  (-4.3%) | -47  (-3.2%) | | |  |
| Rural (nonmetro) | 181 | | 177 | 187 | -5  (-2.5%) | +6  (3.3%) | | 72 | | | 60 | 53 | -12  (-16.1%) | -19  (-25.9%) | | |  |
| ^1^Percent Change = (2020 (or 2021) N – 17-19 Average N)/ 17-19 Average N*100 ^2^NH = Non-Hispanic  ^3^AI/AN = American Indian (Native American)/Alaskan Native ^4^NH/PI = Native Hawaiian/Pacific Islander | | | | | | | | | | | | | | | | |  |

Supplemental Table 3. Rates of suicide and firearm suicide in California from 2017-2021, by sociodemographic characteristics

|  |  | | **All methods** | | | | |  | |  | **Firearm** | | | | |  | | |
| --- | --- | --- | --- | --- | --- | --- | --- | --- | --- | --- | --- | --- | --- | --- | --- | --- | --- | --- |
|  | | | **Suicides per 100,000** | | | | **Rate change (% Change^1^)** | | | **Suicides per 100,000** | | | | | **Rate change  (% Change^1^)** | | | |
|  | | | **2017-19 Average** | | **2020** | **2021** | **2017-19 average  to 2020** | **2017-19 average  to 2021** | | **2017-19 Average** | | | **2020** | **2021** | **2017-19 average  to 2020** | **2017-19 average  to 2021** | | |
| **STATE-WIDE** | | | 11.4 | | 10.5 | 10.4 | -0.9  (-7.9%) | -1.0  (-8.8%) | | 4.1 | | | 3.9 | 4.0 | -0.2  (-4.9%) | -0.1  (-2.4%) | | |
| **SEX** | | | | | | | | | | | | | | | | | |  |
| Female | | | 5.0 | | 4.6 | 4.4 | -0.4  (-8.0%) | -0.6  (-12.0%) | | 0.8 | | | 0.9 | 0.8 | +0.1  (12.5%) | 0  (0.0%) | | |
| Male | | | 17.9 | | 16.4 | 16.5 | -1.5  (-8.4%) | -1.4  (-7.8%) | | 7.5 | | | 7.0 | 7.2 | -0.5  (-6.7%) | -0.3  (-4.0%) | | |
| **AGE GROUP** | | | | | | | | | | | | | | | | | |  |
| 10 to 19 | | | 4.0 | | 4.4 | 4.1 | +0.4  (10.0%) | +0.1  (2.5%) | | 1.1 | | | 1.1 | 1.2 | 0 (0.0%) | +0.1  (9.1%) | | |
| 20 to 29 | | | 12.7 | | 11.7 | 12.0 | -0.9  (-7.1%) | -0.6  (-4.8%) | | 3.5 | | | 3.4 | 4.0 | -0.1  (-2.9%) | +0.5  (14.3%) | | |
| 30 to 44 | | | 12.6 | | 12.5 | 13.3 | -0.1  (-0.8%) | +0.7  (5.6%) | | 3.5 | | | 3.7 | 3.9 | +0.2  (5.7%) | +0.4  (11.4%) | | |
| 45 to 64 | | | 16.0 | | 13.0 | 12.3 | -3.0  (-18.8%) | -3.7  (-23.1%) | | 5.4 | | | 4.5 | 4.3 | -0.9  (-16.7%) | -1.1  (-20.4%) | | |
| 65+ | | | 16.7 | | 16.3 | 16.1 | -0.4  (-2.4%) | -0.6  (-3.6%) | | 9.5 | | | 9.8 | 9.3 | +0.3  (3.2%) | -0.2  (-2.1%) | | |
| **RACE/ETHNICITY** | | | | | | | | | | | | | | | | | |  |
| Hispanic | | | 6.2 | | 6.1 | 6.5 | -0.1  (-1.6%) | +0.3  (4.8%) | | 1.7 | | | 1.6 | 2.0 | -0.1  (-5.9%) | +0.3  (17.7%) | | |
| NH^2^ White | | | 19.4 | | 17.0 | 16.4 | -2.4  (-12.4%) | -3.0 (-15.5%) | | 8.2 | | | 7.6 | 7.4 | -0.6  (-7.3%) | -0.8  (-9.8%) | | |
| NH Asian | | | 6.7 | | 6.8 | 6.6 | +0.1  (+1.5%) | -0.1 (-1.5%) | | 1.3 | | | 1.5 | 1.3 | +0.2  (15.4%) | 0  (0.0%) | | |
| NH Black | | | 8.7 | | 9.8 | 9.8 | +1.1  (12.6%) | +1.1  (12.6%) | | 2.8 | | | 3.6 | 3.8 | +0.8  (28.6%) | +1.0  (35.7%) | | |
| NH AI/AN^3^ | | | 18.2* | | 11.4* | 22.8* | -6.8 (-37.4%) | +4.6 (25.3%) | | 5.3* | | | 4.6* | 4.6* | -0.7  (-13.2%) | -0.7  (-13.2%) | | |
| NH NH/PI^4^ | | | 12.5* | | 11.1* | 9.6* | -1.4 (-11.2%) | -2.9  (-23.2%) | | 4.4* | | | 4.4* | 4.4* | 0  (0.0%) | 0  (0.0%) | | |
| Other | | | 8.1 | | 7.5 | 8.3 | -0.6  (-7.4%) | +0.2  (2.5%) | | 2.4 | | | 2.4 | 1.9* | 0  (0.0%) | -0.5  (-20.8%) | | |
| **HIGHEST LEVEL OF EDUCATION** | | | | | | | | | | | | | | | | | |  |
| Less than a Bachelor’s degree | | | 19.1 | | 17.4 | 17.4 | -1.8  (-9.4%) | -1.7  (-8.9%) | | 6.9 | | | 6.5 | 6.8 | -0.4 (-5.8%) | -0.1 (-1.5%) | | |
| Bachelor's Degree or higher | | | 11.3 | | 10.3 | 10.3 | -0.7  (-6.2%) | -1.0  (-8.9%) | | 4.1 | | | 4.1 | 3.7 | 0 (0.0%) | -0.4  (-9.8%) | | |
| **URBANICITY** | | | | | | | | | | | | | | | | | | |
| Urban (metro) | | | 11.1 | | 10.1 | 10.1 | -0.9  (-8.5%) | -1.0  (-9.2%) | | 3.8 | | | 3.6 | 3.7 | -0.2  (-4.5%) | -0.1  (-3.4%) | | |
| Rural (nonmetro) | | | 21.6 | | 20.6 | 21.8 | -1.0  (-4.8%) | +0.2 (0.9%) | | 8.5 | | | 7.0 | 6.2 | -1.6  (-18.2%) | -2.4  (-27.8%) | | |
| ^1^Percent Change = (2020 (or 2021) Rate – 17-19 Average Rate)/ 17-19 Average Rate*100 ^2^NH = Non-Hispanic  ^3^AI/AN = American Indian (Native American)/Alaskan Native ^4^NH/PI = Native Hawaiian/Pacific Islander  *Rate is based on counts of 30 or less. | | | | | | | | | | | | | | | | | | |

Supplemental Table 4. Proportion of suicides that involved a firearm in California from 2017-2021, by sociodemographic characteristics

|  | Proportion of deaths by suicide that involved a firearm | | | Proportion Change^1^ | |
| --- | --- | --- | --- | --- | --- |
|  | **2017-19 Average** | **2020** | **2021** | **2017-19 average to 2020** | **2017-19 average to 2021** |
| State-wide | 36.1% | 37.6% | 38.1% | 1.5% | 2.0% |
| Sex | | | | | |
| Female | 16.3% | 19.2% | 17.9% | 2.9% | 1.6% |
| Male | 41.7% | 42.8% | 43.5% | 1.1% | 1.8% |
| Age group | | | | | |
| 10 to 19 | 28.9% | 25.8% | 28.3% | -3.1% | -0.6% |
| 20 to 29 | 27.4% | 29.4% | 33.1% | 2.0% | 5.7% |
| 30 to 44 | 28.1% | 29.5% | 29.6% | 1.4% | 1.5% |
| 45 to 64 | 33.9% | 34.7% | 35.2% | 0.8% | 1.3% |
| 65+ | 57.0% | 60.2% | 58.2% | 3.2% | 1.2% |
| Race/ethnicity | | | | | |
| Hispanic | 26.9% | 25.7% | 30.0% | -1.2% | 3.1% |
| NH^2^ White | 42.2% | 44.9% | 45.4% | 2.7% | 3.2% |
| NH Asian | 19.1% | 22.4% | 19.5% | 3.3% | 0.4% |
| NH Black | 32.6% | 37.1% | 39.0% | 4.5% | 6.4% |
| NH AI/AN^3^* | 29.2% | 40.0% | 20.0% | 10.8% | -9.2% |
| NH NH/PI^4^* | 35.3% | 40.0% | 46.2% | 4.7% | 10.9% |
| Other | 29.9% | 31.2% | 23.3%***** | 1.3% | -6.6% |
| Highest level of education | | | | | |
| Less than a  Bachelor’s degree | 36.3% | 37.6% | 39.1% | 1.3% | 2.8% |
| Bachelor's Degree or higher | 36.2% | 38.6% | 36.0% | 2.4% | -0.2% |
| URBANICITY | | | | | |
| Urban (metro) | 34.4% | 35.9% | 36.5% | 1.5% | 2.1% |
| Rural (nonmetro) | 39.5% | 34.0% | 28.3% | -5.5% | -11.2% |
| ^1^Proportion Change = (2020 (or 2021) proportion – 17-19 proportion)  ^2^NH=Non-Hispanic ^3^AI/AN=American Indian  (Native American)/Alaskan Native ^4^NH/PI = Native Hawaiian/Pacific Islander *Proportion is based on counts of 30 or less. | | | | | |

Supplemental Table 5. Counts and rates of suicide and firearm suicide in California from 2017-2021, by month

|  | **Number of suicides (Rate per 100,000)** | | | **Count change (% Change^1^)** | | **Rate change  (% Change^2^)** | |
| --- | --- | --- | --- | --- | --- | --- | --- |
|  | **2017-19 Average** | **2020** | **2021** | **2017-19 average  to 2020** | **2017-19 average  to 2021** | **2017-19 average  to 2020** | **2017-19 average  to 2021** |
| **ALL METHODS** |  |  |  |  |  |  |  |
| January | 366 (0.93) | 382 (0.97) | 337 (0.85) | +16 (4.4%) | -29 (-7.9%) | +0.04 (4.3%) | -0.08 (-8.6%) |
| February | 325 (0.83) | 331 (0.84) | 321 (0.81) | +6 (1.9%) | -4 (-1.2%) | +0.01 (1.2%) | -0.02 (-2.4%) |
| March | 374 (0.95) | 357 (0.91) | 355 (0.9) | -17 (-4.6%) | -19 (-5.1%) | -0.04 (-4.2%) | -0.05 (-5.3%) |
| April | 359 (0.91) | 311 (0.79) | 335 (0.85) | -48 (-13.4%) | -24 (-6.7%) | -0.12 (-13.2%) | -0.06 (-6.6%) |
| May | 376 (0.96) | 305 (0.78) | 377 (0.96) | -71 (-18.9%) | +1 (0.3%) | -0.18 (-18.8%) | 0 (0.0%) |
| June | 403 (1.02) | 355 (0.9) | 364 (0.92) | -48 (-11.9%) | -39 (-9.7%) | -0.12 (-11.8%) | -0.1 (-9.8%) |
| July | 387 (0.98) | 384 (0.98) | 344 (0.87) | -3 (-0.8%) | -43 (-11.1%) | 0 (0.0%) | -0.11 (-11.2%) |
| August | 420 (1.07) | 387 (0.99) | 366 (0.93) | -33 (-7.9%) | -54 (-12.9%) | -0.08 (-7.5%) | -0.14 (-13.1%) |
| September | 373 (0.95) | 330 (0.84) | 364 (0.92) | -43 (-11.5%) | -9 (-2.4%) | -0.11 (-11.6%) | -0.03 (-3.2%) |
| October | 380 (0.97) | 364 (0.93) | 351 (0.89) | -16 (-4.2%) | -29 (-7.6%) | -0.04 (-4.1%) | -0.08 (-8.3%) |
| November | 337 (0.86) | 344 (0.88) | 323 (0.82) | +7 (2.1%) | -14 (-4.2%) | +0.02 (2.3%) | -0.04 (-4.7%) |
| December | 334 (0.85) | 317 (0.81) | 337 (0.85) | -17 (-5.1%) | +3 (0.9%) | -0.04 (-4.7%) | 0 (0.0%) |
| **FIREARM** |  |  |  |  |  |  |  |
| January | 138 (0.35) | 166 (0.42) | 132 (0.33) | +28 (20.3%) | -6 (-4.4%) | +0.07 (20.0%) | -0.02 (-5.7%) |
| February | 124 (0.32) | 127 (0.32) | 117 (0.3) | +3 (2.4%) | -7 (-5.7%) | 0 (0.0%) | -0.02 (-6.3%) |
| March | 142 (0.36) | 137 (0.35) | 139 (0.35) | -5 (-3.5%) | -3 (-2.1%) | -0.01 (-2.8%) | -0.01 (-2.8%) |
| April | 132 (0.34) | 117 (0.3) | 121 (0.31) | -15 (-11.4%) | -11 (-8.3%) | -0.04 (-11.8%) | -0.03 (-8.8%) |
| May | 125 (0.32) | 112 (0.29) | 135 (0.34) | -13 (-10.4%) | +10 (8.0%) | -0.03 (-9.4%) | +0.02 (6.3%) |
| June | 138 (0.35) | 139 (0.35) | 135 (0.34) | +1 (0.7%) | -3 (-2.2%) | 0 (0.0%) | -0.01 (-2.9%) |
| July | 144 (0.37) | 158 (0.4) | 135 (0.34) | +14 (9.7%) | -9 (-6.3%) | +0.03 (8.1%) | -0.03 (-8.1%) |
| August | 149 (0.38) | 132 (0.34) | 138 (0.35) | -17 (-11.4%) | -11 (-7.4%) | -0.04 (-10.5%) | -0.03 (-7.9%) |
| September | 141 (0.36) | 107 (0.27) | 135 (0.34) | -34 (-24.1%) | -6 (-4.3%) | -0.09 (-25.0%) | -0.02 (-5.6%) |
| October | 132 (0.34) | 131 (0.33) | 129 (0.33) | -1 (-0.8%) | -3 (-2.3%) | -0.01 (-2.9%) | -0.01 (-2.9%) |
| November | 121 (0.31) | 117 (0.3) | 127 (0.32) | -4 (-3.3%) | +6 (5.0%) | -0.01 (-3.2%) | +0.01 (3.2%) |
| December | 127 (0.32) | 113 (0.29) | 138 (0.35) | -14 (-11.0%) | +11 (8.7%) | -0.03 (-9.4%) | +0.03 (9.4%) |

^1^Percent Change = (2020 (or 2021) N – 17-19 Average N)/ 17-19 Average N*100 ^2^Percent Change = (2020 (or 2021) Rate – 17-19 Average Rate)/ 17-19 Average Rate*100
